# Recruitment of parvalbumin and somatostatin interneuron inputs to adult born dentate granule neurons

**DOI:** 10.1038/s41598-020-74385-2

**Published:** 2020-10-16

**Authors:** Christine L. Remmers, Charlotte C. M. Castillon, John N. Armstrong, Anis Contractor

**Affiliations:** 1 Department of Physiology, Feinberg School of Medicine, Northwestern University, 303 E. Chicago Ave, Chicago, IL 60611 USA; 2 Department of Neurobiology, Weinberg College of Arts and Sciences, Northwestern University, Evanston, IL 60208 USA

**Keywords:** Neuroscience, Physiology

## Abstract

GABA is a key regulator of adult-born dentate granule cell (abDGC) maturation so mapping the functional connectivity between abDGCs and local interneurons is required to understand their development and integration into the hippocampal circuit. We recorded from birthdated abDGCs in mice and photoactivated parvalbumin (PV) and somatostatin (SST) interneurons to map the timing and strength of inputs to abDGCs during the first 4 weeks after differentiation. abDGCs received input from PV interneurons in the first week, but SST inputs were not detected until the second week. Analysis of desynchronized quantal events established that the number of GABAergic synapses onto abDGCs increased with maturation, whereas individual synaptic strength was constant. Voluntary wheel running in mice scaled the GABAergic input to abDGCs by increasing the number of synaptic contacts from both interneuron types. This demonstrates that GABAergic innervation to abDGCs develops during a prolonged post-mitotic period and running scales both SST and PV synaptic afferents.

## Introduction

Adult-born neurons in the dentate gyrus (DG) of the hippocampus are continuously integrated into hippocampal circuits throughout life, receiving early afferent innervation from local circuit interneurons^1,2^. GABAergic synapses are the first to form onto developing abDGCs and due to their relatively depolarized Cl^-^ reversal potential, GABA is excitatory or shunting during the first 3 weeks post-mitosis^1,3^. Phasic and tonic GABA_A_ receptor activation has been shown to play an important role in progenitors and post-mitotic neurons contributing to proliferation^4^, survival^5^, dendritic development^5^, and formation and unsilencing of glutamatergic synapses onto post-mitotic neurons^6^. Therefore, understanding when and how abDGCs are innervated by GABAergic connections is important for understanding how they integrate into mature hippocampal circuits.

The dentate gyrus contains a diverse population of interneurons which can be classified by their neurochemical expression profile, morphology, and location within the dentate^7,8^. Retrograde transynaptic tracing has demonstrated that somatostatin (SST) and parvalbumin (PV) expressing interneurons synapse onto 3–5-week-old abDGCs^9^ and cholecystokinin (CCK) expressing interneurons synapse onto 5–7-week-old abDGCs^10^. In addition, functional inhibitory connections have been established between abDGCs and PV interneurons 4 and 10 days after differentiation^5,11^, SST interneurons 3 weeks after differentiation^12^, molecular layer performant path (MOPP) interneurons 3–4 weeks after differentiation^9^ as well as Ivy/neurogliaform cells^13^ in POMC-GFP labeled abDGCs that are approximately 2-weeks-old^14^. Despite this there has not been a more detailed analysis of development of GABAergic afferents onto abDGCs during the first few weeks after differentiation.

Two of the most abundant populations of local GABAergic interneurons in the dentate gyrus are PV and SST expressing cells^15^. Both of these types of interneurons have been demonstrated to be synaptically connected to abDGCs^5,9,12^, however the timing of formation of these synaptic contacts in immature abDGCs and how they are modulated by activity during the first 4 weeks following mitosis has not been extensively mapped. Understanding the temporal pattern of development of GABAergic afferents during this early post-mitotic period is important as it coincides with the time at which GABA plays an important role in abDGC survival and functional maturation. PV-expressing interneurons are basket cells that form synapses primarily onto the soma and axon initial segment of mature DGCs^7^. SST interneurons in the dentate are primarily classified as hilar perforant path-associated (HIPP) cells with somata in the hilus and axons projecting through the granule cell layer (GCL) to form synapses onto the dendrites of mature DGCs in the molecular layer^7,16^. Given the distinct location of their axonal projections it is possible that PV and SST interneurons within the dentate form synapses differentially onto abDGCs as they migrate into the granule cell layer and extend their dendrites into the molecular layer.

Here we used optogenetic activation of PV and SST expressing interneurons combined with retroviral birthdating of abDGCs to assess the temporal pattern of development of GABAergic synapses onto immature neurons during the first 4 weeks after differentiation. PV innervation of abDGCs was apparent in the first post-mitotic week, at which point there were no detectable functional SST inputs to abDGCs. The GABAergic input to abDGCs from both PV and SST interneurons increased between 2 and 4 weeks through an increase in synaptic contacts rather than an increase in strength of individual synapses. Voluntary wheel running increased, in parallel, the GABAergic input from both PV and SST interneurons with no effect on the strength of individual synapses and no acceleration in the sequence of input formation. Taken together there is a sequential and systematic increase of GABAergic inputs from PV and SST interneurons onto immature developing abDGCs during the first 4 weeks after differentiation which can be scaled by voluntary wheel running.

## Materials and methods

### Ethics statement

All procedures related to the care and treatment of animals were conducted in accordance with the US National Institutes of Health guidelines for animal research and approved by the Northwestern University Institutional Animal Care and Use Committee (IACUC).

### Animals

PV^Cre^ (Jax stock #017320)^17^ and SST^Cre^ (Jax stock #013044)^18^ mice were crossed with Ai32 (RCL-ChR2(H134R)/EYFP) mice (Jax stock #024109)^19^ (PV-ChR2, SST-ChR2) or Ai9 (RCL-tdT) (Jax stock #007909)^20^ (PV-tdTom, SST-tdTom). All the lines were maintained on a C57Bl/6 background and were propagated by breeding females heterozygous for *Cre* recombinase and homozygous for the Ai32 allele with homozygous Ai32 males. Tail biopsies were used to perform genotyping of all mice used in the study.

### Retroviral birthdating

A replication incompetent retrovirus based on MMLV (Moloney Murine Leukemia Virus) expressing RFP was prepared as described^21,22^. Briefly, GP2-293 packaging cells (Clontech) were co-transfected with p-CAG-RFP and p-CMV-vsv-g (plasmids were a gift from Fred H. Gage at the Salk Institute) using Lipofectamine 2000 (Invitrogen)^21,23,24^. The media was collected from transfected cells 3- and 6-days post-transfection, filtered, and centrifuged at 25,000 rpm to precipitate the virus^21,23,24^. 6–8 week old PV-ChR2 and SST-ChR2 mice of either sex were anesthetized using ketamine/xylazine and 1 μl of virus was injected bilaterally into the hilar region of the dentate gyrus (from Bregma: 0.24 mm posterior, 0.16 mm lateral, 0.24 mm ventral) at a rate of ~ 0.3 μl/min^23^.

### Slice preparation and electrophysiology

Slicing and electrophysiology were carried out as previously described^23^. Briefly, 250 μm coronal slices were prepared at 7, 14, 21, and 28 (± 1) days post retrovirus injection. Fluorescence targeted recordings were made from RFP-expressing neurons in the inner granule cell layer. The composition of the intracellular solution used for voltage clamp recording was (in mM): 95 CsF, 25 CsCl, 10 Cs-HEPES, 10 Cs-EGTA, 2 NaCl, 2 Mg-ATP, 10 QX-314, 5 TEA-Cl, and 5 4-AP, pH adjusted to 7.3 with CsOH. For asynchronous release, slices were perfused with oxygenated artificial cerebrospinal fluid (ACSF) containing 6 mM SrCl_2_, 1 mM MgCl_2_ and 0.5 mM CaCl_2_. Data were collected and analyzed using pClamp 10 software (Molecular Devices). Neurons were voltage-clamped at − 70 mV to record IPSCs. Inclusion of D-APV (50 μM) and CNQX (10 μM) ensured isolation of GABAergic events. Bicuculine (10 μM) was added after recording of optoIPSCs to confirm that events were GABAergic. MiniAnalysis (Synaptosoft) was used to analyze asynchronous events (aIPSCs). ChR2-expressing interneurons were photoactivated using a 5 ms pulse of LED light (470 nm) to the entire visual field. Input–output curves indicate that 500 mV into the LED driver (~ 350 μW of light measured at the slice) consistently evoked the maximal IPSC amplitude in postsynaptic mature and birthdated DGCs and that the response plateaus at higher stimulation intensities (Supplementary Fig. 1). Mature, unlabeled cells were recorded from the outer third of the GCL as most abDGCs have been demonstrated to migrate only to the inner third with only ~ 10% migrating to the outer third of the GCL^25^.

### Running wheels

4–5-week-old PV-ChR2 and SST-ChR2 mice of either sex were single housed with running wheels (MedAssociates) for 3 weeks prior to retroviral injection and were returned to the cage with a running wheel until they were sacrificed for experiments [7, 14, 21 or 28 (± 1) days]. Total distance run was quantified for each day apart from during postoperative quarantine, however animals continued to have access to wheels during this period (Supplementary Fig. 3). Animals ran an average of 7.8 km per day.

### Immunohistochemistry

6–8 week old PV-ChR2 and SST-ChR2 mice of either sex were anesthetized using ketamine/xylazine and perfused with PBS containing 0.02% sodium nitrite and 4 mM MgSO_4_ followed by 2% paraformaldehyde in 0.1 M sodium acetate buffer (pH 6.5) for 6 min, and 2% paraformaldehyde in 0.1 M sodium borate buffer (pH 8.5) for 12–18 min^26,27^. The brains were removed from the skull, post-fixed overnight in 2% paraformaldehyde in 0.1 M sodium borate buffer (pH 8.5), then sectioned coronally at 50 μm on a Leica Vibratome VT1000s in PBS. The sections were collected and stored at 4 °C in PBS containing 0.02% sodium azide. Free-floating sections were rinsed for 30 min in PBS followed by four 15-min washes in PBS containing 0.1% Triton X-100 (Sigma, T8787) and 0.1% bovine serum albumin (BSA; Sigma, A4503). The sections were then blocked with 3% normal donkey serum (NDS; Jackson ImmunoResearch, 017-000-121) for 1 h and then incubated in primary antibody overnight (Rabbit anti-Somatostatin, Peninsula Laboratories, T-4103; mouse anti-parvalbumin, Swant Swiss Antibodies, 235, goat anti-doublecortin (DCX) Santa-Cruz, 8066, chicken anti-NeuN Millipore, ABN91, rabbit anti-RFP AbCam, ab62341). Sections were washed three times for 15 min and then incubated in secondary antibody (donkey anti-guinea pig IgG biotin-SP, Millipore A1938; Streptavidin-Alexa Fluor 680, Invitrogen S32358; donkey anti-rabbit IgG (H + L) Alexa Fluor Plus 594, Invitrogen A33754; donkey anti-goat IgG (H + L) AlexaFluor Plus 680) for 1 h and washed three times prior to mounting and imaging with a 20× objective on a Nikon A1 confocal microscope. Z-series were taken with a 0.82 μm step size.

### Data analysis

OptoIPSCs were analyzed using Clampfit (Molecular Devices) and aIPSCs were analyzed using MiniAnalysis (Synaptosoft). Additional data analysis was conducted with Microsoft Excel and OriginPro software. Comparisons were made with a Mann–Whitney *U* test using OriginPro. Differences were considered significant when p < 0.05. All data are reported as mean ± SEM.

## Results

### Development of PV and SST inputs to abDGCs

**Figure 1:**
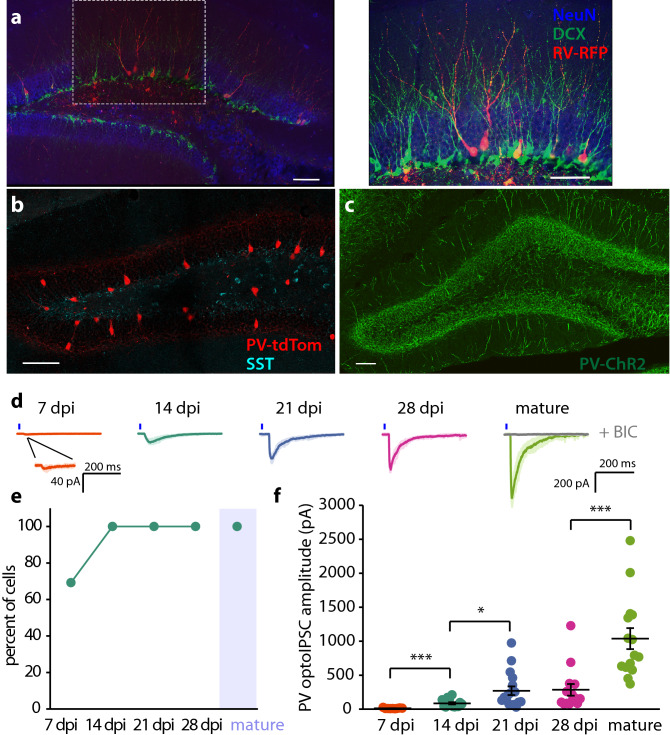
Development of PV inputs to abDGCs during the first 4 weeks after differentiation. (**a**) Image of the dentate gyrus from mice injected with modified retrovirus expressing RFP (RV-RFP) (Left panel) and stained with anti-RFP, anti-NeuN (to label neurons) and anti-DCX (to label young abDGCs localized to the SGZ). Right panel confocal image at high magnification of RFP labeled abDGCs at 21dpi, DCX positive cells (green) and NeuN (blue) (**b**) Confocal image of PV interneurons from PV-tdTom mice demonstrating a lack of colocalization of endogenous SST with TdTom. (**c**) Dendritic and axonal labeling by ChR2-YFP expression in PV interneurons from PV-ChR2 mice. (**d**) Representative traces from whole cell recordings of abDGCs at 7, 14, 21, 28 dpi, and mature DGCs showing the optoIPSCs evoked by photoactivation of PV interneurons. Blue line indicates 5 ms light pulse. Gray trace in mature cell shows effect of 10 μM bicuculine on the optoIPSC response. (**e**) Percent of recorded cells with optoIPSC response to photostimulation of PV interneurons. (**f**) Average amplitudes of optoIPSC in abDGCs at each post-mitotic timepoint. (Error bars represent SEM. *p < 0.05, ***p < 0.001, Mann–Whitney *U*).

**Figure 2:**
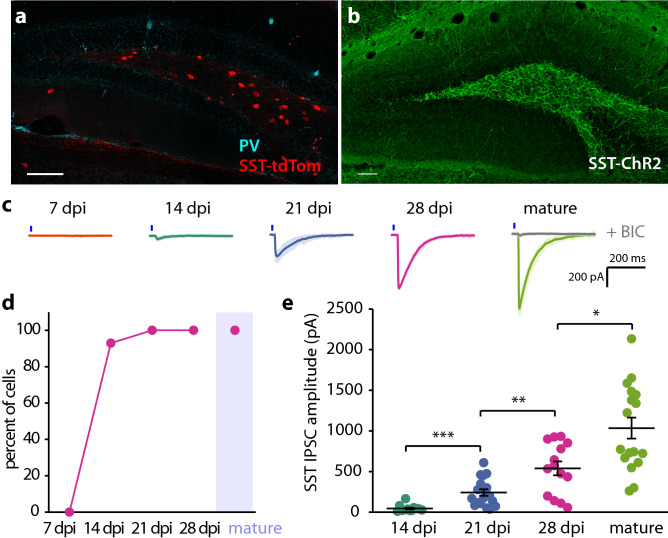
Development of SST inputs to abDGCs during the first 4 weeks after differentiation. (**a**) Confocal image of SST interneurons in SST-tdTom mice demonstrates no colocalization of endogenous PV with tdTom. (**b**) ChR2-YFP expression in SST interneurons from SST-ChR2 mice highlights the dendritic and axonal localization of ChR2. (**c**) Representative traces of whole cell recordings abDGCs at 7, 14, 21, 28 dpi, and mature DGCs illustrating optoIPSCs evoked by photostimulation of SST interneurons. Blue line indicates 5 ms light pulse. Gray trace in mature cell shows effect of 10 μM bicuculine on the optoIPSC response. (**d**) Percent of recorded cells with optoIPSC responses. (**e**) Average optoIPSC amplitude at each day post differentiation (Error bars represent SEM. *p < 0.05, **p < 0.01, ***p < 0.001, Mann–Whitney *U*).

Using PV-ChR2 and SST-ChR2 mice we studied the functional development of GABAergic synapses from these two interneuron types to abDGCs. We first confirmed that PV^Cre^ and SST^Cre^ mice had Cre recombinase expression that reflected endogenous expression of PV and SST by crossing these mice to Ai9 (RCL-tdT) mice to label Cre expressing interneurons. Immunolabeling of PV-tdTom mice for SST and immunolabeling of SST-tdTom mice for PV demonstrated that Cre expression was primarily distinct in the two interneuron populations (Figs. 1b, 2a) as has been previously reported^28^. In addition, expression of YFP in PV^Cre^ mice crossed with Ai32 (RCL-ChR2(H134R)/EYFP)(PV-ChR2) mice clearly demonstrated a pattern of axonal and dendritic labelling of PV interneurons enriched in the GCL consistent with their known patterns of innervation (Fig. 1c)^7,8^. In SST^Cre^ mice crossed with Ai32 mice (SST-ChR2), YFP expression showed a distinct pattern in which the fluorescence was observed in the hilar and molecular regions consistent with the known axonal plexus of SST interneurons (Fig. 2b)^7,8^. Thus, the two mouse lines express Cre recombinase consistent with the expression of neurochemically defined populations of interneurons.

To identify and birthdate newborn DGCs, we injected a modified retrovirus expressing RFP (RV-RFP) into the dentate gyrus of 6–8 week old PV-ChR2 or SST-ChR2 mice. Representative labeled neurons at 21 dpi are visible in the dentate GCL and have the morphology of GCs (Fig. 1a). Notably there was not significant co-labeling with doublecortin (DCX) that primarily labels abDGCs less than 21 days that are localized to the subgranular zone (SGZ) (Fig. 1a)^22^. Full field LED illumination (5 ms) of the slice with the abDGC centered in the field of view illuminated an area approximately 500 μm in diameter. We generated input–output curves by increasing the power of illumination and in all successive experiments the LED power was set to ensure photoactivation of GABA release was maximal (Supplementary Fig. 1). By recording from RFP labeled abDGCs at different timepoints (days post injection, dpi) we measured the macroscopic GABA current in neurons at 7, 14, 21, and 28 dpi and in mature, unlabeled DGCs from the outer granule cell layer^29^. Optogenetic activation of PV interneurons with a single pulse of light elicited an inhibitory postsynaptic current (optoIPSC) in 69.2% of 7 dpi abDGCs (n = 13, 6 cells, animals respectively), and 100% of cells at later timepoints (Fig. 1d,e). The optoIPSC amplitude in PV-ChR2 mice increased over the first 3 weeks following differentiation but plateaued between 21 and 28 dpi before increasing further in mature, unlabeled DGCs (Fig. 1d,f, Table 1). No postsynaptic response to activation of SST interneurons was detected in any of the abDGCs we recorded at 7 dpi (n = 9, 4), but the same stimulation elicited an optoIPSC in 92.9% (n = 14, 6) of 14 dpi abDGCs and 100% of cells recorded at later timepoints (Fig. 2c,d). The optoIPSC amplitude increased as abDGCs matured, indicating an increase in input from SST interneurons during the first 4 weeks of development of abDGCs (Fig. 2c,e, Table 1).

**Table 1:** OptoIPSC amplitude.

	PV control	PV runner	SST control	SST runner
7 dpi	11.6 ± 2.2 pA, n = 13, 6	46.5 ± 10.4 pA, n = 12, 3*	0 ± 0 pA, n = 9, 4	0 ± 0 pA, n = 13, 2
14 dpi	85.6 ± 16.6 pA, n = 13, 4	183.4 ± 27.1 pA, n = 17, 3**	44.6 ± 11.2 pA, n = 14, 6	134.2 ± 33.4 pA, n = 16, 3**
21 dpi	270.2 ± 63.6 pA, n = 21, 6	475.7 ± 97.5 pA, n = 16, 4*	241.2 ± 39.9 pA, n = 18, 5	465.1 ± 62.6 pA, n = 19, 3**
28 dpi	285 ± 85.5 pA, n = 14, 3	811 ± 108.4 pA, n = 10,3***	539.3 ± 84.8 pA, n = 14, 4	871.3 ± 120 pA, n = 16, 4*
mature	1037.5 ± 154.1 pA, n = 15, 6	1099.6 ± 172.9 pA, n = 12, 7	1034.6 ± 129.3 pA, n = 17, 8	988.7 ± 81.9 pA, n = 13, 10

Data are mean ± SEM, n = cells, animals.

Mann–Whitney *U* comparing controls to runners.

*p < 0.05.

**p < 0.01.

***p < 0.001

A comparison of the IPSC responses from PV and SST interneurons provided some interesting contrasts (Supplementary Fig. 2). That we were able to evoke an IPSC in 7 dpi abDGCs in PV-ChR2 mice, but not SST-ChR2 mice indicates abDGCs receive input from PV interneurons prior to SST interneurons (Supplementary Fig. 2a,b). This is consistent with a previous study that found that optogenetic activation of SST interneurons did not evoke an IPSC in 4 dpi abDGCs, but activation of PV interneurons did elicit an IPSC^5^. At 14 dpi, the optoIPSC amplitude was larger in response to activation of PV than SST interneurons (Supplementary Fig. 2c, Table 1). But at 28 dpi the optoIPSC amplitude was larger in response to activation of SST interneurons, indicating that developing abDGCs receive more input from PV interneurons early in development, and that SST input grows rapidly around 3 weeks after differentiation.

### Quantal aIPSCs evoked from SST and PV interneurons

**Figure 3:**
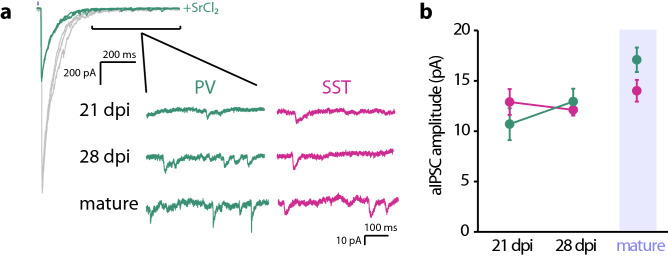
Age dependent increase in PV and SST responses in abDGCs is due to an increase in the number of functional synapses. (**a**) Representative traces show asynchronous IPSCs evoked by optogenetic activation of ChR2-expressing SST and PV interneurons recorded in Sr^2+^ and reduced Ca^2+^. Blue line indicates 5 ms light pulse. Grey trace shows response in normal Ca^2+^. Bracket indicates region analyzed for aIPSCs. (**b**) Amplitude of aIPSCs in abDGCs in SST-ChR2 and PV-ChR2 mice (Error bars represent SEM*).*

To determine whether the average size of individual synaptic connections from the two interneuron types was also scaling with development we analyzed optogenetically-evoked asynchronous IPSCs (aIPSCs) by adding strontium chloride to the extracellular solution to desynchronize GABA release (Fig. 3a)^30^. We recorded aIPSCs at 21 and 28 dpi and in mature DGCs, aIPSC frequency was too low for reliable quantification of events at 14 dpi. We did not observe a significant change in aIPSC amplitude across ages of abDGCs or between recordings from PV-ChR2 and SST-ChR2 mice, indicating that the potency of individual synapses is relatively constant during this time (Fig. 3a,b, Table 2). This also indicates that the increase in amplitude of optoIPSCs across these same time points results from an increasing number of synaptic inputs from both PV and SST interneurons as abDGCs mature. Using these results we were able to calculate an estimate of the number of functional synapses formed from each interneuron type onto developing abDGCs (synapse number ~ optoIPSC amplitude/aIPSC amplitude) (approximate number of PV synapses 21 dpi: 25.3, 28 dpi: 22.0, mature: 65.4) (approximate number of SST synapses 21 dpi: 18.7, 28 dpi: 51.1, mature: 73.8). Moreover, as the quantal amplitude of both PV and SST neurons is not different this confirms that the significantly larger amplitude of optoIPSCs in abDGCs in SST-ChR2 mice compared to PV-ChR2 mice at 28 dpi can be interpreted as increased prevalence of SST inputs at that time (PV: ~ 22.0 synapses; SST: ~ 44.5 synapses).

**Table 2:** aIPSC amplitude.

	PV control	PV runner	SST control	SST runner
21 dpi	10.7 ± 1.6 pA, n = 10, 6	11.9 ± 0.9 pA, n = 10, 5	12.9 ± 1.3 pA, n = 8, 5	10.8 ± 0.6 pA, n = 10, 5
28 dpi	12.9 ± 1.3 pA, n = 10, 5	16.8 ± 1.9 pA, n = 11, 5	12.1 ± 0.6 pA, n = 8, 5	12.9 ± 1.3 pA, n = 9, 5
mature	15.9 ± 1 pA, n = 10, 5	15.5 ± 1.7 pA, n = 10, 5	14 ± 0.7 pA, n = 10, 7	13.7 ± 1 pA, n = 9, 6

Data are mean ± SEM, n = cells, animals.

### Effect of running on PV and SST innervation of abDGCs

**Figure 4:**
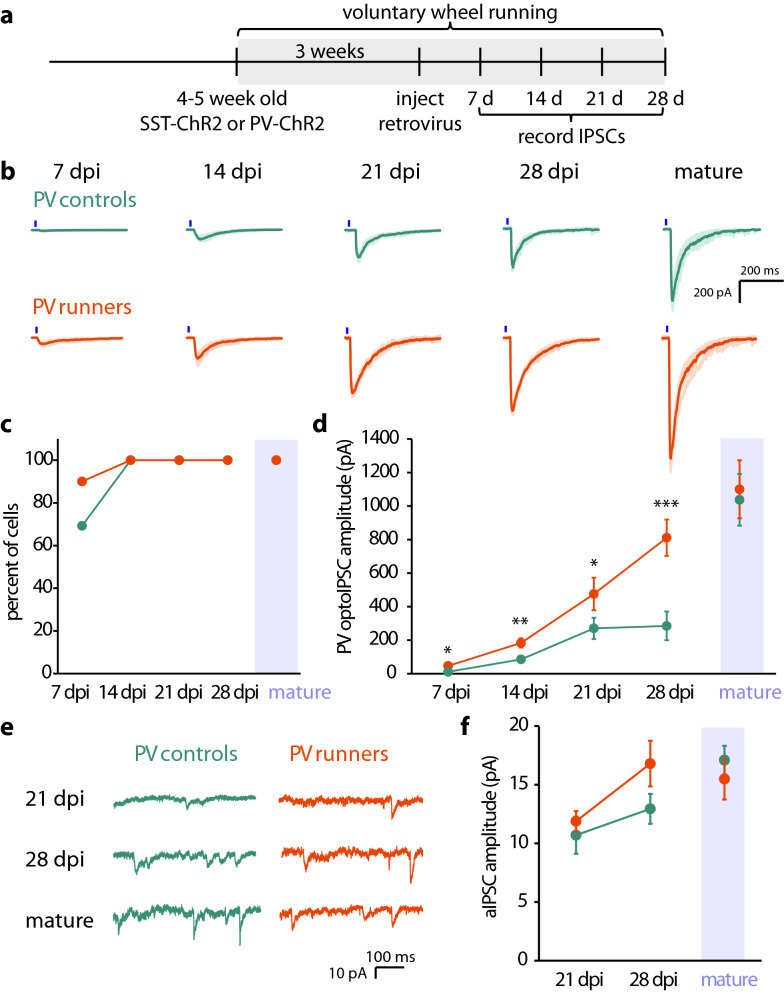
Running scales PV-inputs to abDGCs. (**a**) Timeline of voluntary wheel running experiments. (**b**) Representative traces show optoIPSCs recorded in PV-ChR2 control mice or runners. Blue line indicates 5 ms light pulse. (**c**) Percent of recorded cells with an optoIPSC response to activation of PV interneurons. (**d**) Average optoIPSC amplitude in controls and runners at each timepoint. (**e**) Representative traces show aIPSCs 500 ms trace in PV-ChR2 controls and runners. (**f**) Average amplitude of aIPSCs in abDGCs in PV-ChR2 controls and runners (Error bars represent SEM. *p < 0.05, **p < 0.01, ***p < 0.001, Mann–Whitney *U).*

Voluntary wheel running alters adult hippocampal neurogenesis and improves performance on tasks that rely on intact adult hippocampal neurogenesis^31–35^. However, whether voluntary running also leads to changes in functional maturation of abDGCs is not known. We asked whether the development of PV and SST inputs to abDGCs during the first 4 weeks of maturation, when GABA is critical for neuronal development, is affected when mice are given access to running wheels. PV-ChR2 or SST-ChR2 mice were single housed with running wheels beginning 3 weeks prior to surgery and were then injected with retrovirus to label newborn neurons when mice were 6–8 weeks old (Fig. 4a). We then performed targeted patch-clamp recordings from RFP-expressing cells at 7, 14, 21, and 28 dpi and recorded optogenetically-evoked IPSCs as described above. Mice ran an average of 7.8 km/day, consistent with what has been previously reported in C57Bl/6 mice^36^ (Supplementary Fig. 3).

A greater number of recorded neurons at 7 dpi had functional inputs from PV interneurons in mice that had undergone voluntary wheel running (7 dpi control: 69.2%, n = 13, 6 to PV runner: 90%, n = 12, 3) (Fig. 4b,c). Running scaled the amplitude of optoIPSCs in abDGCs at all dpi recorded, but interestingly had no effect on PV input to mature DGCs (Fig. 4d, Table 1).

**Figure 5:**
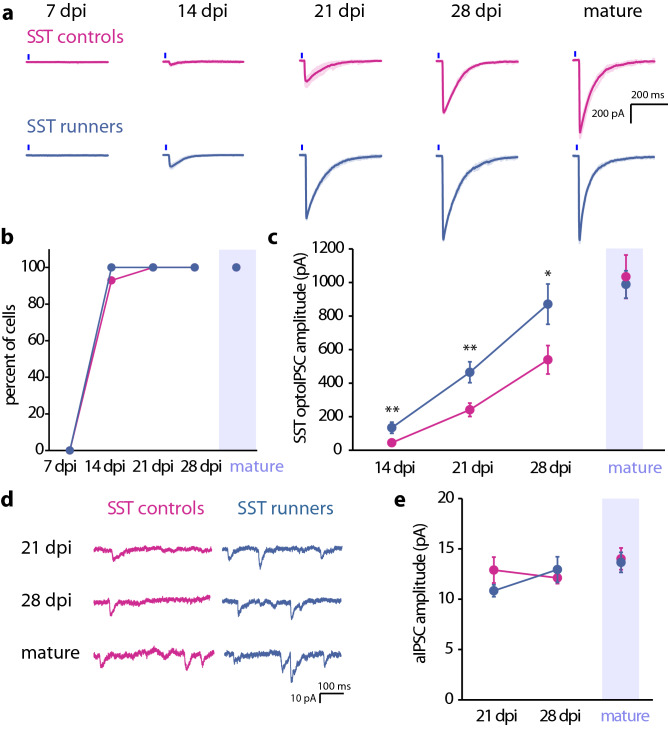
Running scales SST-inputs to abDGCs without accelerating their development. (**a**) Representative traces show SST stimulated optoIPSCs in abDGCs from control mice or runners. Blue line indicates 5 ms light pulse. (**b**) Percent of recorded cells with an optoIPSC response to activation of SST interneurons. (**c**) Average optoIPSC amplitude in controls and runners at each timepoint. (**d**) Representative traces show SST stimulated aIPSCs in controls and runners. (**e**) Average amplitude of SST stimulated aIPSCs in abDGCs in controls and runners (Error bars represent SEM. *p < 0.05, **p < 0.01, Mann–Whitney *U).*

Wheel running failed to accelerate the formation of SST inputs to abDGCs at the earliest timepoints and there were no SST responses in any recorded cells at 7 dpi as with the non-runner group (7 dpi control: n = 9, 4 and 7 dpi runner: n = 12, 2). At 14 dpi responses were found in nearly all recorded cells [92.9% of controls (n = 14, 6) and 100% of runners (n = 16, 3)] (Fig. 5b). As with the PV inputs, running scaled the SST optoIPSC amplitude at all developmental timepoints in abDGCs. Recordings from abDGCs from runner mice demonstrated a larger SST optoIPSC amplitude than recordings from controls (Fig. 5a,c, Table 1). Interestingly, running normalizes the age-dependent differences in inputs from specific interneuron types observed at 14 and 28 dpi in controls. These findings indicate that running scales the GABAergic input that developing abDGCs receive from both PV and SST interneurons, but voluntary wheel running had no detectable effect on interneuron inputs to mature DGCs.

To determine whether running also affected the quantal amplitude of PV and SST synapses we again used strontium replacement to record desynchonized optogenetically-evoked aIPSCs from control mice and runners. We found that access to running wheels had no effect on the average quantal amplitude of IPSC from either PV or SST interneurons in 21 dpi, 28 dpi and mature GCs (Figs. 4e,f, 5d,e, Table 2). These results indicate that the potency of individual inhibitory synapses is not affected by running and that the scaling of the macroscopic GABA currents from SST and PV neurons to abDGCs reflects an increase in the number of synaptic contacts in mice that undergo voluntary wheel running which is evident from calculating the ratio of the average macroscopic current to quantal amplitude for each dpi (PV 21 dpi control: 25.3; 21 dpi runner: 40.0; PV 28 dpi control: 22.0; 28 dpi runner: 48.3; PV mature control: 65.4; mature runner: 71.0; SST 21 dpi control: 18.7; 21 dpi runner: 42.9; SST 28 dpi control: 44.5; 28 dpi runner: 67.3; SST mature control: 73.8; mature runner: 72.4).

## Discussion

Adult born granule cells have intrinsic properties that make them more excitable than mature GCs, yet they remain only sparsely active because of their reduced excitatory synaptic innervation^37^. GABA synapses are the first to be established on post-mitotic DGCs as early as a few days after differentiation^5^. GABA is important for survival, dendritic development, glutamatergic synaptogenesis, and due to the relatively depolarized reversal potential, GABA can induce firing of young abDGCs^3,38^.

While the importance of GABAergic input to the development of very young abDGCs is clear, a better understanding of how abDGCs are innervated during the first 4 weeks after differentiation will help us get a better idea of how these cells integrate into the mature hippocampal circuitry and how they contribute to hippocampal function. Here, we focused on determining the synaptic inputs to abDGCs from two prevalent interneuron types in the dentate gyrus and determined how running activity of the animals modified these connections. As expected, the optoIPSC amplitude from stimulation of both PV and SST interneurons increased across the first 4 weeks of maturation and was even larger in mature DGCs. Confirming the early input from PV interneurons^5^, we found PV mediated responses in a majority of abDGCs at the earliest post-mitotic time we tested (7 dpi), at which point there was no detectable contribution of SST interneurons. Comparison of the amplitudes of the optoIPSC from PV and SST interneurons demonstrated that PV interneurons made the largest contribution at 14 dpi, but at 28 dpi the maximal input from SST interneurons is larger than that from the PV cells. We found that the strength of individual synapses was relatively constant across development, and this allowed us to estimate the number of synaptic inputs from both SST and PV interneurons as abDGCs mature. While our study was ongoing, a study from Groisman and colleagues used a similar approach to map GABA input from PV and SST interneurons onto abDGCs^12^. That study focused on later timepoints after differentiation and concluded that full maturation does not occur until 8 weeks post-differentiation. Moreover, that study did not measure the true quantal size of GABAergic afferents and concluded that the increase in macroscopic currents over abDGC development resulted from scaling of individual synapses rather than synapse number. Our focus on earlier post-mitotic timepoints compared both the macroscopic and true quantal stimulated events from PV and SST inputs and demonstrates that there is an increase in the number of synaptic inputs from each of these interneurons but the relative contribution changes as abDGCs mature.

Adult neurogenesis is a highly regulated process that can be influenced by genetic factors, age, environment, and activity. Voluntary wheel running in mice increases both proliferation and survival of abDGCs, leading to an overall increase in the number of newborn neurons in the dentate gyrus^34^. Running also improves performance on pattern separation and spatial learning tasks, indicating a functional change in the hippocampal circuitry following running^31–33^. However, it is worth noting that there may be no causal relationship between neurogenesis and the running mediated effects on anxiety and learning^39,40^. For instance, one recent study found environmental enrichment (including running wheels) affected spatial learning even after irradiation and ablation of adult hippocampal neurogenesis^41^. So, while the behavioral effects of running may be due to an increased number of newborn neurons, they could also reflect additional alterations in the maturation and integration of abDGCs in mice that have been housed with a running wheel. Voluntary wheel running leads to increased dendritic length and branching in 7 and 16–17 day-old abDGCs and increased density of dendritic spines in 21-day-old abDGCs^42–44^. Running also increases seizure-induced expression of the immediate early gene Arc in 21-day-old abDGCs, indicating that running promotes the integration of newborn neurons into the existing hippocampal circuit^45^. Functionally, running reduces the input resistance and increases the percentage of abDGCs with GABAergic input in 7 dpi abDGCs in rats^46^ and increased and the number of NMDA only synapses in 7 dpi abDGCs in mice^43^. Interestingly, the number of anatomically traced presynaptic neurons to abDGCs was not affected by running, which together with our finding that running increases the optoIPSC amplitude suggests that the primary effect of running is to increase the number of synaptic contacts per presynaptic neuron onto very young abDGCs^43^. On the other hand, in 5-week-old abDGCs running decreased connectivity between local hippocampal neurons without affecting mIPSC frequency or amplitude^47^. Thus, there may be differential age-dependent effects on connectivity between abDGCs and local circuit interneurons that are caused by running. The present study included animals of both sexes, however it is worth noting that proliferation and survival of abDGCs may be differentially modulated by stress and learning in males and females^48^.

Our approach enabled us to focus on the inputs to abDGCs from two defined interneuron types and perform a comprehensive analysis of the effects of running on functional connectivity of abDGCs during the first 4 weeks after differentiation. We found that there was a scaling of all GABA inputs to abDGCS from both SST and PV interneurons, but quantal size did not scale in parallel. The most parsimonious explanation for these results would be that the number of synaptic inputs to abDGCs (rather than the potency of individual synapses) scales after voluntary wheel running. Given that previous studies found no effect of running on the numbers of connected local interneurons this might suggest that individual axons form more synaptic connections with abDGCs in mice that are runners. Future tracing studies with high resolution neuroanatomy would be required to confirm this conclusion. Interestingly we found no effect of running on GABA input to mature cells. A recent study used a Fos-Trap approach to identify mature granule cells that are active during a single 2-h bout of running and found considerable plasticity of their excitatory inputs from the entorhinal cortex^49^. In our analysis we were not able to specifically identify DGCs that might have been active during long term running, but it is possible that PV and SST inputs might be differentially modulated in active DGCs compared to the total population.

PV interneurons are the most well-characterized source of GABA inputs to abDGCs. GABA release from PV interneurons maintains quiescence of adult neural stem cells (aNSCs) while in vivo optogenetic or chemogenetic activation of PV interneurons increases the survival and promotes dendritic growth in abDGCs^5,11,50^. PV interneurons in the dentate are primarily basket cells with somata in the granule cell layer and axons forming perisomatic synapses onto mature DGCs^7,8^. Much less is known about SST inputs to abDGCs, although Song and colleagues reported that they were unable to detect SST inputs to less than 1-week-old abDGCs using optogenetic activation^5^. SST interneurons in the dentate are primarily HIPP cells that form synapses on the distal dendrites of mature DGCs^7,8^. Therefore, it is likely that the temporal sequence of innervation from these distinct populations of interneurons is determined in part by the laminar organization of interneuron axons in the dentate gyrus. At the earliest timepoint, when dendrites have not extended beyond the granule cell layer, abDGCs receive perisomatic input from PV interneurons with axons in the granule cell layer. As the abDGCs mature and extend their dendrites into the molecular layer, they encounter the axons of SST interneurons and form functional synapses.

In summary, we characterized the temporal sequence of development of inputs from SST and PV interneurons onto abDGCs during the first 4 weeks after differentiation and determined that voluntary wheel running scales these GABAergic connections. Our data are consistent with an age and activity dependent increase in the number of functional synapses from both PV and SST interneurons which could reflect an increase in the number of synapses formed per axon with an individual abDGC. Voluntary running does not accelerate the process of connectivity as there was no shift in when abDGCs receive SST input. Together these studies further our understanding of the how abDGCs mature and are integrated into the hippocampal network.

## Supplementary information

Supplementary Legends.

Supplementary Figure 1.

Supplementary Figure 2.

Supplementary Figure 3.

Supplementary Table 1.
