# Supplementary material for: Recruitment of parvalbumin and somatostatin interneuron inputs to adult born dentate granule neurons
Source: Sci Rep. 2020 Oct 16;10:17522. doi: 10.1038/s41598-020-74385-2 (PMC7568561; doi:10.1038/s41598-020-74385-2)
Supplement: Supplementary file 1 — Supplementary Legends. [file 41598_2020_74385_MOESM1_ESM.docx]

**Supplementary Figure 1. LED power to photoactivation current recorded in abDGCs. a-d)** Input-output curves show normalized optoIPSC amplitude in response to a 5 ms pulse of LED light with power into the LED driver varying from 0-800 mV in mature (a) or 28 dpi abDGCs (**b**) recorded in PV-ChR2 mice and mature (**c**) or 28 dpi abDGCs (d) recorded in SST-ChR2 mice. Arrows at indicate voltage that consistently evoked the maximal IPSC amplitude and was used in all subsequent experiments (500 mV into the LED driver, ~350 μW of light measured at the slice).

**Supplementary Figure 2.** **Age dependent increase in PV and SST responses in abDGCs. (a)** Representative traces of optoIPSCs recorded in PV-ChR2 and SST-ChR2 mice. Blue line indicates 5 ms light pulse. Gray trace in mature cell shows effect of 10 μM bicuculine on the optoIPSC response. (**b)** Shows the percent of abDGCs in which a light-evoked response was observed in SST-ChR2 and PV-ChR2 mice at each timepoint. (**c)** Comparison of the average optoIPSC amplitude in PV-ChR2 and SST-ChR2 mice at each timepoint.

**Supplementary Figure 3. Distance travelled during voluntary wheel running. (a)** The total distance run per day was tracked for a cohort of mice. Retroviral injection was performed at day 0. Distance run was not quantified during the postoperative quarantine, however animals continued to have access to wheels during this period.
