# Supplementary material for: Recruitment of parvalbumin and somatostatin interneuron inputs to adult born dentate granule neurons
Source: Sci Rep. 2020 Oct 16;10:17522. doi: 10.1038/s41598-020-74385-2 (PMC7568561; doi:10.1038/s41598-020-74385-2)
Supplement: Supplementary file 5 — Supplementary Table 1. [file 41598_2020_74385_MOESM5_ESM.docx]

Supplementary Table 1. OptoIPSC data by sex

|  |  | **Female** | **Male** |
| --- | --- | --- | --- |
| **PV control** | **7 dpi** | 6.8 ± 1 pA, n = 3, 2 | 8.4 ± 0 pA, n = 3, 1 |
|  | **14 dpi** | 68.7 ± 19.3 pA, n = 8, 3 | 112.6 ± 28.7 pA, n = 5, 1 |
|  | **21 dpi** | 255.5 ± 92 pA, n = 8, 3 | 291.2 ± 89.2 pA, n = 7, 4 |
|  | **28 dpi** | 171.3 ± 31.6 pA, n = 9, 2 | 489.7 ± 216.1 pA, n = 5, 1 |
|  | **mature** | 1382.1 ± 365 pA, n = 5, 3 | 861.3 ± 185.7 pA, n = 6, 2 |
| **PV runners** | **7 dpi** | -- | 44.5 ± 11.6 pA, n = 9, 3 |
|  | **14 dpi** | -- | 187.8 ± 28.5 pA, n = 17, 3 |
|  | **21 dpi** | 443.3 ± 123.7 pA, n = 11, 2 | 547 ± 167.6 pA, n = 5, 2 |
|  | **28 dpi** | 1012.6 ± 350.3 pA, n = 3, 1 | 724.6 ± 59.1 pA, n = 7, 2 |
|  | **mature** | 1337.3 ± 211 pA, n = 8, 3 | 624.1 ± 90.2 pA, n = 4, 2 |
| **SST control** | **7 dpi** | 0 ± 0 pA, n = 9, 4 | -- |
|  | **14 dpi** | 22.5 ± 5.3 pA, n = 5, 2 | 61.5 ± 18.8 pA, n = 7, 2 |
|  | **21 dpi** | 230.8 ± 52.9 pA, n = 11, 3 | 257.6 ± 65.1 pA, n = 7, 2 |
|  | **28 dpi** | 439.5 ± 102.5 pA, n = 10, 2 | 917.8 ± 58.4 pA, n = 6, 2 |
|  | **mature** | 940.7 ± 166.3 pA, n = 8, 5 | 1118.1 ± 199.5 pA, n = 9, 3 |
| **SST runners** | **7 dpi** | -- | 0 ± 0 pA, n = 13, 2 |
|  | **14 dpi** | 224.8 ± 73.4 pA, n = 6, 1 | 79.8 ± 17.4 pA, n = 10, 2 |
|  | **21 dpi** | 462.6 ± 73.3 pA, n = 16, 2 | 478.1 ± 89.8 pA, n = 3, 1 |
|  | **28 dpi** | 790.8 ± 150 pA, n = 6, 2 | 901.3 ± 195.8 pA, n = 11, 2 |
|  | **mature** | 852.6 ± 90.5 pA, n = 6, 3 | 895.1 ± 266.3 pA, n = 2, 2 |
